# Probiotic Effects on Multispecies Biofilm Composition, Architecture, and Caries Activity *In Vitro*

**DOI:** 10.3390/microorganisms8091272

**Published:** 2020-08-21

**Authors:** Zhihui Chen, Sebastian Schlafer, Gerd Göstemeyer, Falk Schwendicke

**Affiliations:** 1 Department of Oral Diagnostics, Digital Health and Health Services Research, Charité-Universitätsmedizin, 14197 Berlin, Germany; zhihui.chen@charite.de; 2 Department of Dentistry and Oral Health, Aarhus University, 8000 Aarhus, Denmark; sebastians@dent.au.dk; 3 Department of Operative and Preventive Dentistry, Charité-Universitätsmedizin Berlin, 14197 Berlin, Germany; gerd.goestemeyer@charite.de

**Keywords:** biofilm model, demineralization, dental caries, enamel, transverse microradiography

## Abstract

While probiotics have been tested for their anti-caries effect in vitro and also clinically, there is a lack of understanding of their effects on complex dental biofilms. We assessed two probiotics, *Lactobacillus reuteri* and *Streptococcus oligofermentans*, on a continuous-cultured model containing *Streptococcus mutans*, *Lactobacillus rhamnosus* and *Actinomyces naeslundii*. Cariogenic biofilms were grown on bovine enamel specimens and daily challenged with *L. reuteri* or *S. oligofermentans* whole culture (LC/SC) or cell-free supernatant (LS/SS) or medium only (negative control, NC) (*n* = 21/group) for 10 days. Biofilm was assessed via counting colony-forming units, quantitative polymerase chain reaction, and fluorescence in situ hybridization. Caries activity was determined by pH measurements and by assessing mineral loss (ΔZ) using transverse microradiography. Both LC and SC significantly reduced total and strain-specific cariogenic bacterial numbers (*p* < 0.05). ΔZ was reduced in LC (mean ± SD: 1846.67 ± 317.89) and SC (3315.87 ± 617.30) compared to NC (4681.48 ± 495.18, *p* < 0.05). No significant reductions in bacterial numbers and ΔZ was induced by supernatants. Biofilm architecture was not considerably affected by probiotic applications. Viable probiotics *L. reuteri* and *S. oligofermentans*, but not their culture supernatants, could reduce the caries activity of multi-species biofilms *in vitro*.

## 1. Introduction

Caries is a multifactorial disease characterized by an imbalance (“dysbiosis”) in the dental biofilm composition and activity, triggered by available fermentable carbohydrates allowing cariogenic (i.e., acidogenic and aciduric) bacterial strains to metabolize these to organic acids. The resulting pH decrease generates an ecological advantage for pathogens and results in mineral loss from dental hard tissues [1].

Probiotics have been described as microorganisms that provide a health benefit and that have the potential to modify the microflora and re-balance the described dysbiosis [2,3]. However, the association between this modification and any clinically relevant effects on dental caries (lesion prevention or arrest) has not been unambiguously established [4,5,6]. Hence, a range of in vitro studies has been conducted to better understand the effects of widely available probiotic strains on dental pathogens [7,8,9], demonstrating probiotic effects mainly based on three mechanisms—an enhanced activity of the arginine deiminase system (ADS), direct inhibition of pathogens, and indirect reduction of pathogen colonization by competition for surface receptors. ADS activity contributes to pH homeostasis by generating alkali from L-arginine, thereby reducing the ecological advantage of aciduric pathogens while preventing dental hard tissue demineralization [10,11]. Direct inhibition of pathogens has been demonstrated via competing for common nutrients or by inducing the production of inhibitory substances, i.e., bacteriocins and metabolic products, or by co-aggregation with cariogenic organisms [12,13,14]. Additionally, probiotic organisms have been shown to compete for binding sites on surfaces or host cells and thus, exclude the adherence of pathogenic bacteria [15].

*Lactobacillus reuteri* and *Streptococcus oligofermentans* are two probiotic species that have been shown to harness one or more of these strategies: *L. reuteri* exhibits significant antibacterial activity against both periodontopathic and cariogenic bacteria, mainly through reuterin, an antimicrobial component produced by glycerol fermentation [7,16,17,18]. *S. oligofermentans* possesses strong adhesion and low carbohydrate fermentation capacity, and produces hydrogen peroxide by utilizing lactic acid, thereby inhibiting pathogens such as *Streptococcus mutans* [19]. However, there is a paucity of data illustrating how these strains interact not with single pathogens, but with complex multispecies biofilms, which is a better reflection of the clinical situation. This limitation in modeling probiotic effects might explain the gap between promising in vitro results and contradictory clinical data on the anti-caries efficacy of probiotics.

Here, we aimed to employ a validated multi-species cariogenic biofilm model of *S. mutans*, *Lactobacillus rhamnosus*, and *Actinomyces naeslundii* to assess the effects of *L. reuteri* and *S. oligofermentans* on biofilm composition, architecture and caries activity *in vitro*.

## 2. Materials and Methods

### 2.1. Sample Preparation

From the crowns of 50 bovine incisors of the second dentition, without cracks, fractures, caries, or any defects, 105 enamel specimens were prepared (4.5 × 4.5 × 4 mm; band saw EXAKT 300 CL, EXAKT Apparatebau, Norderstedt, Germany). Specimens were ground flat (abrasive paper 1200–2500 grit, Hermes WS FLEX 18C, Buehler, Düsseldorf, Germany) and polished (silicon carbide grinding paper 4000 grit, Buehler). Specimens used for assessing biofilm composition (*n* = 5/group, 25 in total) were covered with nail varnish (Rival de Loop, Rossmann, Burgwedel, Germany) except for the polished enamel surface. Specimens used for assessment of mineral loss and biofilm structure (*n* = 16/group, 80 in total) were embedded in acrylic resin (Technovit 4071, Heraeus Kulzer, Hanau, Germany) before polishing, after which approximately one-third of the surface was covered with nail varnish to protect it from biofilm-induced demineralization (sound control). Specimens were then sterilized (121 °C, 2.1 bar, 34 min) and stored at room temperature for further experiment.

### 2.2. Bacterial Strains and Culture Conditions

*Lactobacillus reuteri* ATCC PTA 5289 and *Streptococcus oligofermentans* DSM 8249 (BioGaia, Stockholm, Sweden) were employed as probiotic strains; *Lactobacillus rhamnosus* DSM 20021, *Streptococcus mutans* DSM 20523, *Actinomyces naeslundii* DSM 43013 (Leibniz Institute DSMZ-German Collection of Microorganisms and Cell Cultures, Braunschweig, Germany) as cariogenic ones. *Lactobacillus* strains were cultured on de Man-Rogosa-Sharpe agar (MRS agar, Oxoid, Wesel, Germany), all other strains for 48 h on Columbia Agar with 5% Defibrinated Sheep Blood (Thermo Fisher Scientific, Vantaa, Finland). Single colonies were transferred into modified Brain Heart Infusion (MBHI) broth (BHI supplied with 8 g/L meat extract, 1% glucose, 1% sucrose, 250 mM glycerol, and 10 mM L-arginine, pH 7.0; Carl Roth, Karlsruhe, Germany) and incubated for 24 h. Incubation for all experimental steps, including the continuous-culture biofilm model, was conducted at 37 °C under aerobic conditions.

### 2.3. Multispecies Biofilm Model

**Figure 1:**
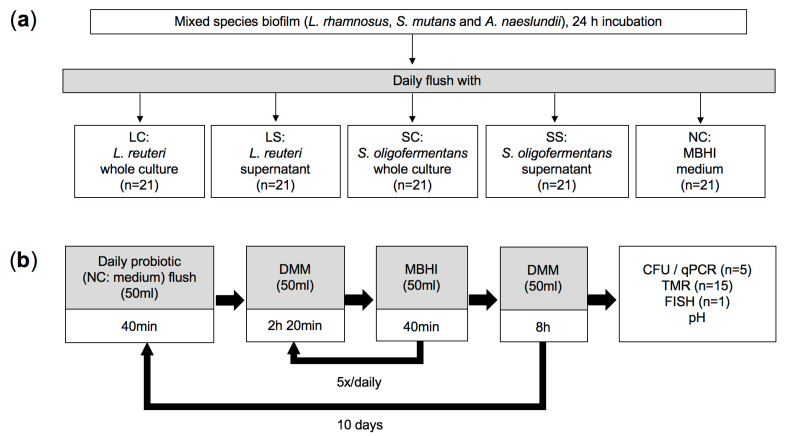
Study flow. (**a**) Multispecies biofilms were cultured for 24 h and then submitted to daily rinses with *L. reuteri* whole culture (LC), *L. reuteri* culture supernatant (LS), *S. oligofermentans* whole culture (SC), *S. oligofermentans* culture supernatant (SS) or fresh MBHI medium (NC). (**b**) The daily provision of modified Brain Heart Infusion (MBHI) broth and Defined Mucin Medium (DMM) was repeated over 10 days before submitting biofilms and specimens to further analysis. CFU: colony-forming units; qPCR: quantitative polymerase chain reaction; TMR: transverse microradiography; FISH: fluorescence in situ hybridization; n: sample size.

The sterilized enamel specimens (*n* = 21/group) were randomly allocated to five experimental groups in different chambers and pre-coated overnight with sterile-filtrated human saliva to allow for pellicle formation. Human saliva was collected from a healthy donor (28-years old female with low caries risk, without any caries, periodontal or systemic disease, who had not received antibiotic therapy for at least three months before the study) following a previously described protocol [20]. After the collection, the saliva was centrifuged (10 min, 6000× *g*) and filter-sterilized by 0.22 μm syringe filter (Millex-GP Syringe Filter, Merck, Darmstadt, Germany). Overnight cultures of the three cariogenic strains were adjusted and diluted to the optimal optical density at 600nm (OD_600_) in fresh MBHI. Fifty-milliliter mixed suspension containing the equal amounts of each strain (approx. 1 × 10^8^ colony-forming units; CFU) was delivered into each chamber to create cariogenic biofilms (Figure 1a).

After 24 h of incubation, the chambers were connected to computer-controlled pumps (ISM405 MCP-Z standard pump with MS/CA pump head, ISMATEC, Wertheim, Germany) as previously described [21]. This continuous-cultured system was then challenged by different experimental interventions: (1) *L. reuteri* whole culture (LC); (2) *L. reuteri* culture supernatant (LS); (3) *S. oligofermentans* whole culture (SC); (4) *S. oligofermentans* culture supernatant (SS) and (5) negative medium control (NC) (Figure 1a). In LC and SC groups, *L. reuteri* or *S. oligofermentans* full cultures in MBHI, collected in the late exponential phase at a concentration of 1 × 10^9^ CFU/mL, were provided for 40 min (5 mL/min, 20 min influx/efflux time, 20 min rest) once per day. In LS and SS groups, sterile-filtrated culture supernatants were provided in the same manner, instead of full cultures. NC group was rinsed with plain MBHI medium instead of full cultures or supernatants. Afterwards, all groups received fresh MBHI for 40 min (5 mL/min, 20 min influx/efflux time, 20 min rest), followed by rinsing (5 mL/min, 20 min influx/efflux time) and resting (2 h) in modified defined mucin medium (DMM; 3 mM KCl, 0.5 mM K_2_HPO_4_, 0.5 mM KH_2_PO_4_, 1 mM NaCl, 0.2 mM NH_4_Cl, 1 mM CaCl_2_ × 2H_2_O, 0.2 mM MgCl_2_ × 6H_2_O, 0.5 mM CO(NH_2_)_2_, 2.5 g/L mucin, pH 7.0). This medium/DMM cycle was repeated five times daily to simulate the consumption of food and saliva clearance. The day was concluded with an 8-h resting period (Figure 1b). After 10 days of cultivation at 100% humidity, samples were removed and processed for analysis at the end of the daily schedule of the final day. The study setup is summed up in Figure 1.

### 2.4. Quantification of Bacterial Numbers via CFU

Following the incubation period, enamel samples were removed and gently washed with 0.9% sterile saline twice. The attached biofilms on the sample surface were carefully collected from identical surfaces (4.5 × 4.5 mm) and transferred into 1 mL sterile saline using a sterile curette. After vortex mixing, 100 μL of the microbial suspensions were plated out in triplicate on Columbia Agar with Sheep Blood (Thermo Fisher Scientific) in serial 10-fold dilutions. Total viable bacteria numbers were determined after incubation for 48 h at 37 °C aerobically.

### 2.5. DNA Isolation and Quantitative Polymerase Chain Reaction (qPCR)

For the DNA extraction from collected biofilms, the collected biofilm suspension (900 μL) was centrifuged (20 min, 6000× *g*), after which the pellet was re-suspended in 500 μL enzyme solution (20 mg/mL lysozyme, 1.2% Triton, 2 mM EDTA, 20 mM Tris-HCl, pH 8.0) and incubated at 37 °C for 1 h. The DNA isolation was performed using the QIAamp mini DNA Extraction Kit (Qiagen, Hilden, Germany) according to the manufacturer’s instructions. Total genomic DNA was quantified by spectrophotometer (Multiskan Microplate Spectrophotometer, Thermo Fisher Scientific) and stored at 20 °C before further processing. The three cariogenic strains were quantified by qPCR as described previously [22]. In brief, strain-specific primers for 16S rRNA gene amplicons were designed using the Primer3 software [23], checked for specificity using BLAST, and synthesized by Metabion (Planegg, Germany). The primers specific for *L. rhamnosus* were forward primer AGGTGCTTGCATCTTGATTT and reverse primer CGCCATCTTTCAGCCAAGAA, and the annealing temperature was 62 °C. The primers specific for *S. mutans* were forward primer GACGCAAGGGAACACACT and reverse primer TCATGCAATAATTAATATTATGCGGTA, and the annealing temperature was 62 °C. The primers specific for *A. naeslundii* were forward primer TTTGTGGGTCCTGGATGAGT and reverse primer AAAAAGGCGCAATCTTTCC, and the annealing temperature was 64 °C. No cross-reactivity of the primers with other participating strains was observed. Quantification of *L. rhamnosus*, *S. mutans*, and *A. naeslundii* in the biofilms was performed by qPCR using a CFX Connect Thermal Cycler with a 96-well reaction module (Bio-Rad, Hercules, United States). Each reaction mixture (final volume 10 μL) contained 1 μL template DNA, 5 μL SYBR Select Master Mix (Applied Biosystems, Vilnius, Lithuania), 2 μL 400 nm forward/reverse primers and 2 μL RNase-free water (Braun Biotech, Melsungen, Germany). The applied thermal profile consisted of an initial denaturation at 95 °C for 2 min, followed by 40 cycles of denaturing at 95 °C for 20 s and annealing for 60 s at the specific annealing temperature. Melting curve analysis was performed for all primer sets to ensure a single peak, which was indicative of primer specificity. All experiments were performed in triplicate. The genomic DNA of the three bacterial species in the unknown samples was quantified using standard curves constructed by 10-fold serial dilution of bacterial DNA, which was extracted from pure cultures in MBHI of each strain collected at early stationary phase, with the optical density adjusted to a concentration of about 1 × 10^9^ CFU/mL. Following the same extraction method described for the qPCR reaction, the DNA content was determined and serially diluted for the generation of standard curves correlating quantification cycle values to CFU per mL range from 1 × 10^4^ to 1 × 10^9^.

### 2.6. Caries Activity Determination (pH Measurements and Transverse Microradiography)

On the final day of the continuous biofilm culture, liquid waste in each chamber was collected and sterile filtrated, respectively, before and after the resting period, i.e., 16 h and 24 h after the last probiotic rinse. The pH value in the liquid was determined in triplicate measurements using a pH-meter (GMH3510 Digital pH-/mV-/Thermometer, Greisinger, Regenstauf, Germany).

For the transverse microradiography (TMR), samples were cut vertically to the surface into thin sections, ground to a mean thickness of 100 ± 10 μm, and polished up to 4000 grit as described. Microradiographs of the thin sections were obtained by a nickel-filtered copper X-ray source operating at 20 kV and 20 mA with an exposure time of 10 s. Films (Fine 71,337, Fujifilm, Tokyo, Japan) were developed according to the manufacturer’s recommendation under standardized conditions. The radiographic images were digitally analyzed using a light microscope connected to CCD-video camera module (XC-77CE, Sony, Tokyo, Japan). A digital image analysis software (TMR 2.0.27.2, Inspektor Research Systems, Amsterdam, Netherlands) was used to analyze the integrated mineral loss (ΔZ, in vol % × μm) and lesion depth (LD, in μm). Enamel areas covered with nail varnish served as sound reference.

### 2.7. Fluorescence In Situ Hybridization (FISH)

Five specimens were randomly selected from each of the five experimental groups and subjected to FISH for a qualitative analysis of the biofilm architecture. The samples were fixated in 3% (*w*/*v*) paraformaldehyde/PBS for at least 16 h, washed three times in PBS for 20 min and then dehydrated in an ethanol series (50%, 60%, 70%, 80%, 90%, 100%) for 1 h each. Then the specimens were transferred to a vacuum chamber (Exsikkator, Kartell S.p.A. LABWARE Division, Noviglio, Italy) and infiltrated with cold polymerizing resin (Technovit 8100, Heraeus Kulzer) for 10 min with the vacuum on, followed by 3 h with the vacuum turned off. The infiltration procedure was performed twice. Samples were then embedded in resin for 16 h using polyethylene embedding molds (Flat Bottom Embedding Capsules, Electron Microscopy Sciences, Hatfield, PA, USA). All of the above procedures were carried out at 4 °C. Before decalcification in 17% EDTA for 21 days, the specimens were cut into thin slices of 1 mm with a rotary saw microtome (Ernst Leitz, Wetzlar, Germany). Decalcification was checked by X-ray analysis, after which the slices were re-embedded in resin (Technovit 8100, Heraeus Kulzer), sectioned on an ultramicrotome (2 µm; Ultracut E, Reichert Jung Optische Werke, Wien, Austria) and mounted on glass slides with adhesive coating (Polysine, Menzel-Gläser, Braunschweig, Germany).

The probes STR405 [24], 5′-labeled with Alexa Fluor 488 (MWG Biotech, Ebersberg, Germany) and ACT476 [25], 5′-labeled with Atto 550 (IBA, Göttingen, Germany) were used to detect streptococci (*S. mutans* and *S. oligofermentans*) and *A. naeslundii*, respectively. Additionally, EUB338 [26], 5′-labeled with Atto 633 (IBA), was employed to visualize the remaining bacteria (*L. rhamnosus* and *L. reuteri*). For each experiment, fixed cells of all strains employed in the study were used as positive and negative controls for the three probes. All probes were specific at 30% formamide in the hybridization buffer (Figure S1). Permeabilization, hybridization, stringency washes, and mounting were performed according to the protocol described previously [27].

After mounting, the biofilms were analyzed by confocal laser scanning microscopy (Zeiss LSM700, Zeiss, Jena, Germany). Dyes were excited sequentially at 488 nm (Alexa 488), 543 nm (Atto 550), and 633 nm (Atto 633) and detected from 300–550 nm (Alexa 488), 560–800 nm (Atto 550) and 300–800 nm (Atto 633). Images were acquired at representative sites with a 63× objective (Plan-Apochromat; NA = 1.4; oil; Zeiss) objective, with the pinhole set to 1 Airy Unit, a pixel time of 1.55 µs and an image size of 1048 × 1048 pixels (101.6 × 101.6 µm^2^).

### 2.8. Statistical Analysis

The statistical analysis was performed with SPSS 25 (IBM, Armonk, Armonk, NY, USA). Normal distribution and homogeneity were checked using Shapiro–Wilk and Levene’s tests, respectively. One-way analysis of variance (ANOVA) followed by Dunnett’s multiple comparisons test for assessing differences between groups regarding the log-transformed CFU data, numbers of cariogenic bacteria quantified by qPCR, pH value, LD and ΔZ. The level of significance was set at *p* < 0.05 for all statistical tests.

## 3. Results

### 3.1. Viable Cell Counts of Biofilms

**Figure 2:**
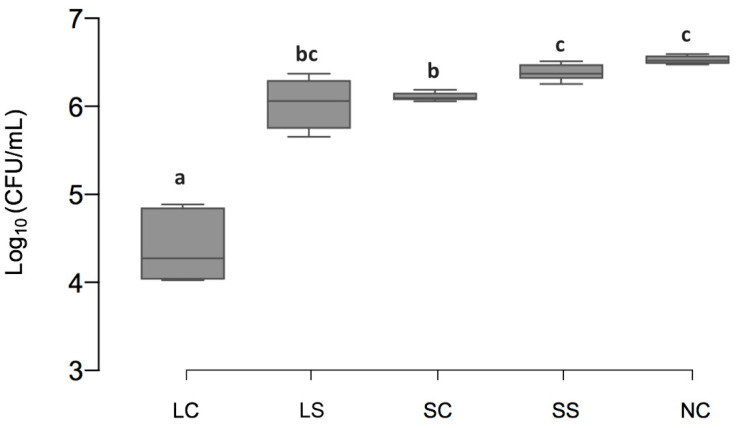
The total amount of viable bacteria. Line and box = median and 25th/75th percentiles; error bars = minimum and maximum. LC: *L. reuteri* whole culture; LS: *L. reuteri* culture supernatant; SC: *S. oligofermentans* whole culture; SS: *S. oligofermentans* culture supernatant; NC: negative medium control. Different superscript letters indicate statistically significant differences between groups (*p* < 0.05); if boxes share minimum one letter there was no significant difference detected (*p* > 0.05).

The total amount of viable bacteria [log_10_ (CFU/mL), mean ± SD] was significantly lower in LC (4.88 ± 0.42) and SC (6.11 ± 0.05, *p* < 0.001) than NC (6.60 ± 0.05, *p* < 0.001). For LS and SS (6.37 ± 0.29 and 6.37 ± 0.10, respectively) no significant difference to NC was observed (*p* > 0.05) (Figure 2).

### 3.2. Cariogenic Bacterial Amounts

In the LC group, *L. rhamnosus* and *S. mutans*, but not *A. naeslundii* were significantly reduced compared with NC (*p* < 0.05). For SC group, *S. mutans* and *A. naeslundii*, but not *L. rhamnosus* were significantly reduced compared with NC (*p* < 0.05). LS and SS did not show any significant differences compared with NC (*p* > 0.05) (Table 1).

### 3.3. pH Value

The pH value in the LC group was significantly higher than that in most other groups, specifically NC, after both 16 and 24 h of the last probiotic rinse, respectively (*p* < 0.05). SC did not provide obvious pH alterations compared with NC. For LS and SS, a significant decrease compared to NC was observed after 24 (but not 16) h (*p* < 0.05) (Table 2).

### 3.4. Mineral Loss of Enamel Lesions

**Figure 3:**
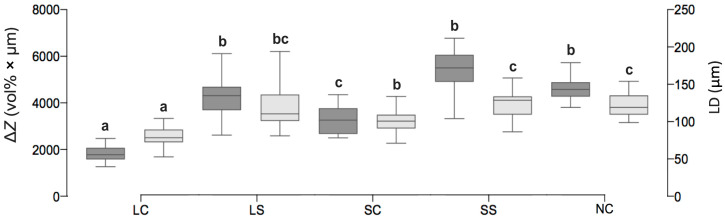
Mineral loss ΔZ (dark grey boxes, left y-axis) and lesion depth LD (light grey boxes, right y-axis) of enamel caries lesions after 10 days of biofilm cultivation. Line and box = median and 25th/75th percentiles; error bars = minimum and maximum. LC: *L. reuteri* whole culture; LS: *L. reuteri* culture supernatant; SC: *S. oligofermentans* whole culture; SS: *S. oligofermentans* culture supernatant; NC: negative medium control. Different superscript letters indicate statistically significant differences between treatment groups (*p* < 0.05); if boxes share minimum one letter there was no significant difference detected (*p* > 0.05).

**Figure 4:**
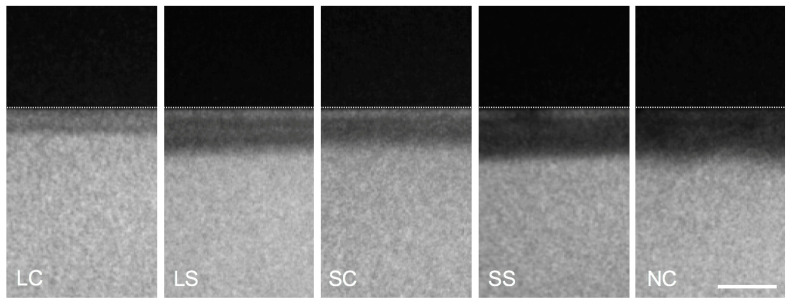
Representative microradiographs of the carious lesions on enamel surface induced by the multispecies biofilms after 10 days. The dotted white line indicates the lesion interface. The mean value of lesion depths differed from 78.2 μm (LC) to 122.4 μm (NC). LC: *L. reuteri* whole culture; LS: *L. reuteri* culture supernatant; SC: *S. oligofermentans* whole culture; SS: *S. oligofermentans* culture supernatant; NC: negative medium control. Scale bar = 100 μm.

ΔZ (vol% × μm, mean ± SD) and LD (μm, mean ± SD) (Figure 3 and Figure 4) was significantly lower in LC (ΔZ: 1846.67 ± 317.89, LD: 78.20 ± 13.13) and SC (3315.87 ± 617.30, 101.35 ± 15.08) than NC (4681.48 ± 495.18, 122.39 ± 15.72, *p* < 0.01). In LS and SS, no significant differences to NC were observed (*p* > 0.05).

### 3.5. Biofilm Structure and Composition

**Figure 5:**
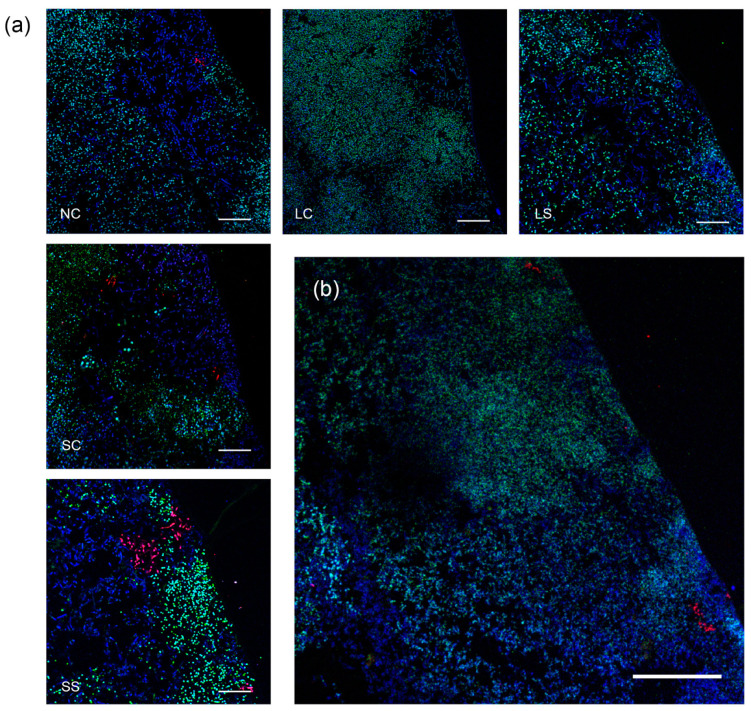
Biofilm architecture, visualized by fluorescence in situ hybridization (FISH). (**a**) In all five treatment groups, compact biofilms were observed, dominated by clusters of streptococci (STR405-Alexa-488, green) and *Lactobacillus spp* (EUB338-Atto-633, blue). *A. naeslundii* (ACT476-Atto-550, red) was identified in low numbers in all biofilms, as branched colonies typically located at the biofilm base. Scale bars = 20 µm. (**b**) Representative image (from LS group) demonstrates the complex biofilm architecture with cell clusters, cell-free areas, and a bulged surface, which resemble the structure of supragingival biofilm. No significant structural modification was observed between experimental groups. Scale bar = 60 µm. Dark areas represent the enamel surface of the specimens. LC: *L. reuteri* whole culture; LS: *L. reuteri* culture supernatant; SC: *S. oligofermentans* whole culture; SS: *S. oligofermentans* culture supernatant; NC: negative medium control.

FISH analysis of biofilms attached to the enamel specimens revealed the presence of compact structures in all treatment groups. The biofilms had a thickness of 150–250 µm and showed typical architectural features of supragingival biofilms with a bulged surface. In all treatment groups, the biofilms were dominated by streptococci and *Lactobacillus* spp. *A. naeslundii* was identified in low numbers in all biofilms, as characteristic branched colonies typically located at the biofilm bases (Figure 5).

## 4. Discussion

Probiotics have been demonstrated as a potentially useful agent for the prevention or treatment of oral diseases. However, data on the efficacy of probiotics on caries prevention and management remains ambiguous [4]. The present study assessed the application of viable *L. reuteri* and *S. oligofermentans* cultures as well as their supernatants on cariogenic multispecies biofilms. We found the vital probiotics to significantly decrease the enamel mineral loss, bacterial numbers, and cariogenic activity of the biofilm. *L. reuteri* tended to show superior anti-caries potential compared with *S. oligofermentans*, while the supernatants exerted no evident beneficial effects.

Our findings can be interpreted from a range of perspectives. First of all, the presence of viable probiotics could significantly affect the composition and caries activity of the biofilm, as reflected by changes in CFU and bacterial numbers, pH and mineral loss. We likely ascribe this contribution to the modification of inherent ADS activity: Evidence from both clinical and laboratory studies suggest a positive relationship between ADS activity and dental health. ADS contributes to pH homeostasis by generating alkali from arginine metabolism, and thereby reduces the ecological advantage of aciduric pathogens, which is grounded in a positive regulation between microflora acidification and aciduric bacterial selection [28,29,30,31]. The arginolytic strains *L. reuteri* and *S. oligofermentans* were able to constantly neutralize acid products via the ADS route and slow down the caries process. In the supernatant groups, without the persistent participation of active probiotic bacteria, biofilms had no opportunity to reverse the disadvantaged pH milieu and hence remained in a pathogenically metabolic pattern. The addition of probiotic microorganisms could prevent further ecological shifts toward disease and improve biofilm resilience [32,33,34,35]. For dysbiotic biofilms, probiotics could offer a chance to reconstruct a host-compatible community, while for healthy ones, to maintain health status and improve the resilience of the ecosystem.

In contrast, any indirect actions of probiotics that have been mediated by supernatant, e.g., bacteriocins (such as reuterin), are likely to play a minor role in the observed microbial communities. This seems to conflict with our previous study, which described reuterin in the supernatant as a promising antimicrobial factor [36]. However, our previous study only assessed *S. mutans* monospecies biofilms. Multi-species biofilm tends to be more aggressive, unite [37,38], and better recover from external shocks, especially those provided only once per day (as in the present study). The polymeric matrix, containing extracellular polymeric substances (EPS) such as polysaccharides, and soluble glucans that are produced by cariogenic pathogens, offers a robust habitat for surface adhesion, synergistic/competitive interactions, and antimicrobial tolerance against external chemical and physical fluctuations, e.g., probiotic bacteriocins [39]. Besides, the polymicrobial synergy could modulate virulence gene expression and therefore elevate the pathogenicity of the bacterial community [40]. Note that we did account for the environment-specific probiotic actions, i.e., certain external conditions required for probiotics to exert their anti-cariogenic talent [41]. Therefore, we supplemented our media with low concentrations of glycerol and arginine, both common nutrients that could be acquired from diet and saliva, to assure that the probiotics share adequate nutrition for correlative metabolic pathways in the simulation system. Glycerol is required by *L. reuteri* to generate reuterin, and arginine is an essential educt for the ADS system [42,43].

Species-specific effects were also observed: *L. reuteri* reduced the bacterial numbers of *L. rhamnosus* and *S. mutans*, but not *A. naeslundii*. *L. reuteri* also exhibited more pronounced capacity than *S. oligofermentans* in modification on the cariogenic activity of biofilms. The latter, however, impacted more on the bacterial numbers of *S. mutans* and *A. naeslundii* rather than *L. rhamnosus*, which may explain the divergence in caries activity reduction between LC and SC. *L. reuteri* probably achieves successful lesion control through the significant inhibition of dominant strains in our complex biofilm model, *L. rhamnosus* and *S. mutans*, which are fast-growing and competitive under severely acidic conditions. Though both probiotic strains inhibit the reproduction of *S. mutans*, *L. reuteri* is suggested via bacteriocin and *S. oligofermentans* via hydrogen peroxide products [7,19], which is also a powerful agent specifically against facultative anaerobes such as *A. naeslundii*. Moreover, the genera streptococci and lactobacilli remained dominant in the biofilms regardless of which probiotic intervention was employed, and biofilm architectures visualized by FISH were largely unaffected. This suggests that, as described, the probiotics had larger intra-genus than inter-genus effects, i.e., *L. reuteri* had mainly replaced *L. rhamnosus* and *S. oligofermentans* mainly replaced *S. mutans*. The congeneric antagonism between *S. mutans* and other common streptococci, such as *S. oligofermentans*, *Streptococcus sanguinis*, and *Streptococcus gordonii* has been well studied [44,45]. The interspecies competition probably exists also between *L. reuteri* and *L. rhamnosus*, which share similar metabolic behavior and, therefore, fight for limited carbohydrate resources in tight-knit communities, which may lead to the starvation of the competing pathogen *L. rhamnosus* [46,47]. This replacement of highly acidogenic species from the same genus is likely to be one of the strategies for probiotic to moderate the dental plaque aggressiveness. From the clinical perspective, the combined application of vital probiotics from both genera, streptococci and lactobacilli, would be a wise choice.

This study has several strengths and limitations. Firstly, and as a strength, we employed a biodiverse and complex caries model to assess the anti-caries effects of both viable cultures and supernatants of *L. reuteri* and *S. oligofermentans.* Our model aims to mimic natural conditions as best as possible while retaining operational feasibility [21,48,49]. Secondly, we have proven the favorable efficacy of the probiotic intervention on the caries process, as the gold standard for assessing mineral loss in vitro [50], TMR, provided direct evidence of the anti-caries potential of the two probiotic strains. A range of outcome parameters (e.g., the biofilm composition, architecture) were also acquired via a diverse set of measures (CFU, qPCR, FISH, pH). For example, species-specific quantification using qPCR allowed us to overcome the limits of differential sensitivity of CFU when enumerating bacteria from the same genus, while CFU is more suitable to reflect the impact of interventions on vital bacteria with metabolic activity. Serial assays with mutual complementation provided us a comprehensive interpretation of biofilm changes under probiotic therapy. Thirdly, as a limitation, our model remained a simplification of natural processes. It does not permit to reflect the impact of more complex and dynamic oral microbiome, host responses, oral hygiene behaviors, or diets on cariogenicity. Additionally, our FISH analysis allowed only for a qualitative assessment of biofilm structure and composition rather than quantification, and also did not focus on cariogenic versus probiotic strains. We accepted that, partially as further methods were employed to obtain information in these directions. Further studies are required to elucidate why supernatant did not, as described, have any significant impact on cariogenic biofilms, and should also assess how environment-specific conditions modify the anti-caries efficacy of two probiotic strains in more detail.

## 5. Conclusions

Within the limitations of this in vitro study, viable probiotics *L. reuteri* and *S. oligofermentans*, but not their culture supernatants, had the potential to reduce the cariogenic effects of multi-species biofilms in vitro. Viable probiotics had species selective effects on cariogenic strains in a complex multispecies biofilm model.

## Figures and Tables

**Table 1:** Bacterial numbers (× 10^6^ cells/mL, mean ± SD) of each cariogenic strain in the different treatment groups, as determined by qPCR (*n* = 5).

Group	*L. rhamnosus*	*S. mutans*	*A. naeslundii*
LC	0.55 ± 0.10 ^a^	0.37 ± 0.11 ^a^	0.19 ± 0.04 ^a^
LS	4.03 ± 1.87 ^ab^	3.65 ± 1.86 ^abc^	0.36 ± 0.20 ^ab^
SC	2.23 ± 0.86 ^ab^	0.22 ± 0.14 ^ab^	0.09 ± 0.04 ^b^
SS	3.84 ± 1.61 ^ab^	2.76 ± 0.57 ^c^	0.43 ± 0.07 ^a^
NC	3.13 ± 0.87 ^b^	3.69 ± 1.38 ^c^	0.45 ± 0.10 ^a^

Different superscript letters indicate the statistically significant difference between experimental groups within each column measured on the same time point (*p* < 0.05); if groups share minimum one letter there was no significant difference detected (*p* > 0.05).

**Table 2:** Final pH values (mean ± SD) of the fluid in different biofilm chambers for five experimental groups 16 and 24 h after the last probiotic rinse.

Group	16 h	24 h
LC	5.00 ± 0.02 ^a^	3.90 ± 0.03 ^a^
LS	3.90 ± 0.02 ^b^	3.53 ± 0.01 ^b^
SC	3.83 ± 0.01 ^cd^	3.75 ± 0.00 ^a^
SS	3.73 ± 0.03 ^d^	3.44 ± 0.01 ^c^
NC	3.94 ± 0.03 ^bc^	3.70 ± 0.01 ^d^

Different superscript letters indicate the statistically significant difference between experimental groups within each column measured at the same time point (*p* < 0.05); if groups share minimum one letter there was no significant difference detected (*p* > 0.05).

## Supplementary Materials

The following are available online at https://www.mdpi.com/2076-2607/8/9/1272/s1, Figure S1: Specificity of the employed FISH probes.
