# Supplementary material for: Probiotic Effects on Multispecies Biofilm Composition, Architecture, and Caries Activity In Vitro
Source: Microorganisms. 2020 Aug 21;8(9):1272. doi: 10.3390/microorganisms8091272 (PMC7565971; doi:10.3390/microorganisms8091272)
Supplement: Supplementary file 1 [file microorganisms-08-01272-s001.pdf]

microorganisms

MDPI

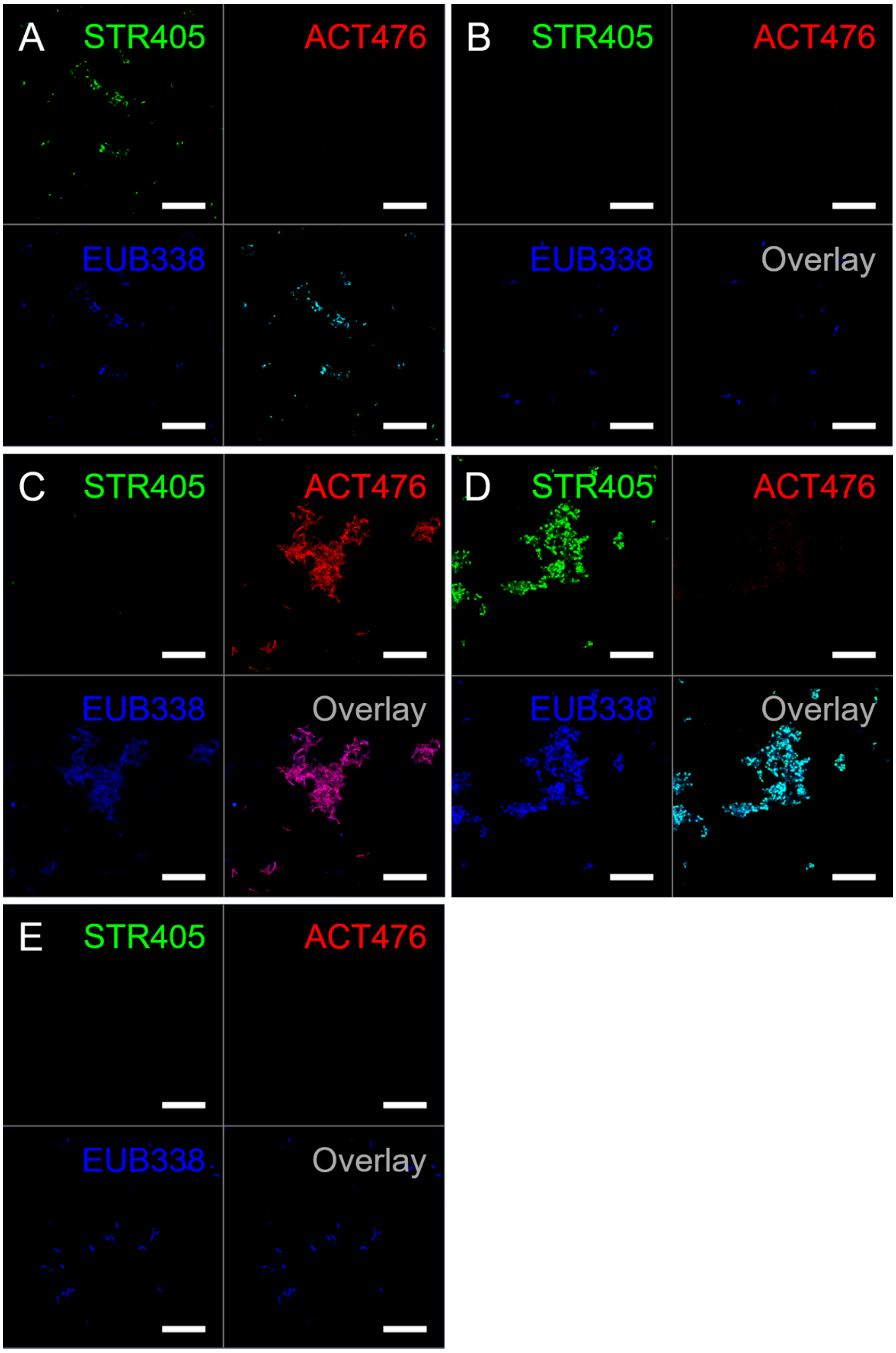

Figure S1. The specificity of the employed FISH probes. At a formamide concentration of 30 % in the hybridization buffer, all probes were specific for their targets. *S. mutans* (**A**) and *S. oligofermentans* (**D**) were visualized by the *Streptococcus*-specific probe STR405 and the universal probe EUB338, but not by the *Actinomyces*-specific probe ACT476. *L. rhamnosus* (**B**) and *L. reuteri* (**E**) were targeted by EUB338 only. *A. naeslundii* (**C**) was visualized by ACT476 and EUB338, but not by STR405. Scale bars = 20 µm.
